# Targeting, out-scaling and prioritising climate-smart interventions in agricultural systems: Lessons from applying a generic framework to the livestock sector in sub-Saharan Africa

**DOI:** 10.1016/j.agsy.2016.05.017

**Published:** 2017-02

**Authors:** An Notenbaert, Catherine Pfeifer, Silvia Silvestri, Mario Herrero

**Affiliations:** a CIAT (International Center for Tropical Agriculture), PO Box 823-00621, Nairobi, Kenya; b WUR (Wageningen University), P.O. Box 430, Wageningen, 6700, AK, The Netherlands; c ILRI (International Livestock Research Institute), PO Box 30709-00100, Nairobi, Kenya; d CABI (Centre for Agriculture and Bioscience International), PO Box 633-00621, Nairobi, Kenya; e CSIRO (Commonwealth Scientific and Industrial Research Organisation), Brisbane, Australia

**Keywords:** Targeting, Priority setting, Climate smart agriculture, Livestock

## Abstract

As a result of population growth, urbanization and climate change, agricultural systems around the world face enormous pressure on the use of resources. There is a pressing need for wide-scale innovation leading to development that improves the livelihoods and food security of the world's population while at the same time addressing climate change adaptation and mitigation. A variety of promising climate-smart interventions have been identified. However, what remains is the prioritization of interventions for investment and broad dissemination.

The suitability and adoption of interventions depends on a variety of bio-physical and socio-economic factors. Also their impacts, when adopted and out-scaled, are likely to be highly heterogeneous. This heterogeneity expresses itself not only spatially and temporally but also in terms of the stakeholders affected, some might win and some might lose. A mechanism that can facilitate a systematic, holistic assessment of the likely spread and consequential impact of potential interventions is one way of improving the selection and targeting of such options.

In this paper we provide climate smart agriculture (CSA) planners and implementers at all levels with a generic framework for evaluating and prioritising potential interventions. This entails an iterative process of mapping out recommendation domains, assessing adoption potential and estimating impacts. Through examples, related to livestock production in sub-Saharan Africa, we demonstrate each of the steps and how they are interlinked. The framework is applicable in many different forms, scales and settings. It has a wide applicability beyond the examples presented and we hope to stimulate readers to integrate the concepts in the planning process for climate-smart agriculture, which invariably involves multi-stakeholder, multi-scale and multi-objective decision-making.

## Highlights

• Planning and decision making processes for CSA are invariably multi-stakeholder, multi-scale and multi-objective in nature.
• An iterative process of mapping domains, assessing adoption potential and estimating impacts helps prioritizing CSA options.
• A multitude of methods can be applied and combined for carrying out these different steps.
• A participatory approach thereby allows for continuous learning and growing levels of trust and buy-in from stakeholders.

## 1 Introduction

The UN Food and Agriculture Organisation (FAO) estimates that by 2050 farmers will have to produce 70% more food to meet the needs of the world's expected population of 9.1 billion people (FAO, 2009). The Intergovernmental Panel on Climate Change (IPCC), on the other hand, is warning that the global climate is changing and expected to continue to do so in the foreseeable future (IPCC, 2014). Agriculture will need to adapt to this looming challenge to maintain food security, economic activities and the livelihoods of many, especially in developing countries (Howden et al., 2007). Agriculture also contributes to climate change (CC), with the agriculture, forestry, and other land use sectors contributing 24% of anthropogenic global greenhouse gas emissions (Smith et al., 2014).

Keating et al. (2014) argued that changes in the agricultural sector are essential and proposed a three-pronged approach across the science and policy domains, complementing actions to increase the food production with interventions that sustain the productive capacity of the food system and others that aim at managing food demand. Around the same time, the Food and Agriculture Organisation of the United Nations (FAO) introduced CSA as an integrative approach to address the interlinked challenges of food security and climate change (FAO, 2013). CSA explicitly aims for three objectives:

• sustainably increasing agricultural productivity, to support equitable increases in farm incomes, food security and development;
• adapting and building resilience of agricultural and food security systems to climate change at multiple levels; and
• reducing greenhouse gas emissions from agriculture (including crops, livestock and fisheries).

CSA incorporates technologies, policies, institutions and investment. It includes on-farm interventions, such as composting, mulching, intercropping, improved animal feeding, integration of drought-tolerant crop varieties and climate-risk insurance, as well as interventions beyond the farm, such as carbon financing, establishing efficient markets and better weather forecasting. It is equally about the issues as it is about the process to go about resolving them.

While a lot of work has been done in the solution space and promising interventions have been identified, one of the main issues remains the selection, targeting and prioritization of interventions. Substantial investments are being made in CSA, but to ensure that the resources are appropriately allocated, all relevant development actors need information on which interventions are suitable and likely to reach the greatest possible positive impact across the different objectives of food and nutrition security, CC mitigation and CC adaptation.

CSA investment decisions are, however, challenged by a three-fold complexity. First, CSA is multi-objective by nature. Ideally, all three goals of CSA would be achieved. In reality, such triple-win solutions are rare and trade-offs between the different objectives are often observed. Specialised farming, for example, might be highly productive, it is also sensitive to changes in climate and thus not satisfying the adaptation criterion. Second, the impacts of CSA interventions vary by scale, both in time and in space. Management decisions made at the household level have effects on the individual components of the household-level system, and can have aggregated effects at village, regional, watershed and landscape level (Klapwijk et al., 2014). These effects at lower or higher scales are often synergistic but this is unfortunately not always the case. Similarly, short term gains are often not sustained in the long term. Last, but not least, a wide range of stakeholders are influencing and/or affected by CSA decisions. These stakeholders' perception on what is important might differ and thus result in conflicting judgements (Nordström et al., 2012). There is thus a need for these different stakeholders to engage in a dialogue and negotiation process.

Starting from the targeting framework described by Herrero et al. (2014), we developed a generic framework for targeting, out-scaling and prioritising CSA interventions in agricultural systems. The methodology entails a multi-stage and iterative process of (1) diagnosis and identification of alternative options, (2) characterisation of the options, (3) identification of the recommendation domains and out-scaling potential of these options, (4) assessing the impacts along different dimensions and on different groups of people. This paper describes how we applied these generic steps to CSA prioritization in livestock production systems in sub-Saharan Africa. We discuss lessons learnt and the implications for research for development.

## 2 Materials and methods

### 2.1 A framework for targeting, scaling out and prioritising interventions in agricultural systems

#### 2.1.1 Introduction

**Fig. 1:**
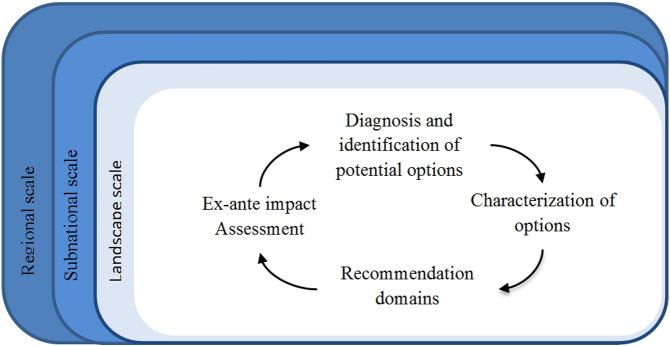
Four generic steps for targeting, scaling out and prioritising interventions in agricultural systems.

The framework for targeting, scaling out and prioritising CSA interventions explicitly integrates systems analysis, targeting and ex-ante impact assessment in the decision-making processes. Through the integration of comprehensive and reliable information as inputs into planning processes, it aims to contribute to informed CSA planning. Its target users are all those that are involved in CSA planning and implementation processes. The framework consists of four generic steps which are explained below (Fig. 1).

Though these four steps follow some logical order and represent the initial workflow, with information from one step feeding into the next step, the process of targeting and prioritising in not a linear task. It rather entails a multi-stage but iterative process with recurrent learning and refining of analysis and results. Going through the process with multiple feed-back and feed-forward loops, both within and between the four stages, allows for an increasingly deeper understanding and -if applied in a truly participatory way- increasing levels of trust and buy-in from stakeholders. Discrepancies between stakeholders' opinions is thereby likely to shift due to change of knowledge or interest (Brandt et al., 2015).

A multitude of participatory approaches that can be drawn upon exist, such as Rapid Rural Appraisal (RRA), Participatory Rural Appraisals (PRA), Participatory Video (PV) and Participatory Mapping (PGIS). In deciding which method to employ, one must take into account (i) the reasons for involvement and expected outcomes, (ii) the nature and scope of the issue, (iii) who is affected, interested or can contribute to solutions, (iv) amount of time available and (v) availability of resources (Slocum, 2003).

#### 2.1.2 Step 1: Diagnosis and identification of potential options

A first step involves ‘diagnosis and identification of potential options’. Depending on the local environment and current problems encountered in the landscape, a different set of interventions is needed. Farmers and livestock keepers face a wide variety of challenges, such as food insecurity, high poverty levels, low and variable yields, declining soil fertility and land degradation. Some of these challenges are wide spread, others can be found in selected locations only. This step consists of a description of the agricultural system in terms of issues and problems encountered as well as specific opportunities and potential solutions that exist. Agricultural systems are very complex; farmers and livestock keepers have differential access to human, financial, physical, natural and social resources and engage in a wide variety of livelihood strategies. In addition, the systems are not static nor do they operate in a vacuum. They influence, and are influenced by, the surrounding environment or context. Policies, norms, institutions, the economic climate and how they are changing thus all need to be taken into account in both the diagnostic and solution space. This step consists of an integrated and participatory process of combining, interpreting and communicating knowledge from diverse scientific disciplines to allow a better understanding of complex phenomena (Rotmans and Van Asselt, 1999). Guiding questions for this step are:

- What are the available resources (human, natural, financial, social and physical) and how are they organised for agricultural production?
- What are the current levels and trends of crop and livestock productivity, demand and consumption? Are there demand, yield or resource gaps that can/need to be addressed? What are appropriate options to address them?
- What are the different climate change scenarios and associated impacts; on the natural resource base as well as on the population? How much uncertainty is associated with these climate and impact scenarios?
- What/who is most vulnerable and why? How can their vulnerability be decreased?
- What are the main sources and sinks of greenhouse gasses (GHGs)? Where is there most potential to mitigate GHG emissions or capture carbon? And which interventions would this take?
- Who are the main stakeholders? Are there likely losers and winners? Who can influence the decision or wider context in which the agricultural production and potential CSA interventions are taking place?

#### 2.1.3 Step 2: Characterisation of options

This step implies careful scrutiny and characterisation of the solutions being offered in Step 1. Due to the wide range of climatic conditions; cultural, institutional, and economic factors; and their interactions, there is an immense diversity of agricultural practices. This means there is a correspondingly large array of possible intervention options (Howden et al., 2007). All proposed interventions, be them at technical, institutional or policy level, are applicable in different settings and under different conditions. They may be hampered by specific barriers to adoption, such as lack of economic feasibility, too high risks involved or poor business planning skills, and require well-targeted supporting incentives. It is thus important to identify for all alternatives those characteristics that affect their use and adoption. These characteristics include bio-physical and socio-economic context variables, but also technology-specific characteristics such as the capital, labour, capacity and land requirements. The second step results in a –quantitatively or qualitatively described- collection of potential options and provides the target users with a menu of un-prioritised choices. Single technologies or practices –even if applied in suitable environments- cannot address the full suite of issues encountered in complex agricultural systems. In many cases different practices have to be combined or “mixed and matched” to identify overall farm- or landscape strategies. Some research programs specifically aim at describing these packages of interventions, through e.g. participatory land-use planning. In that case, potential trade-offs and synergies between the interventions at system or landscape scale will also have to be described.

Different approaches can be used to define the criteria that determine suitability and adoption. A first set of approaches starts from the assumption that these criteria are relatively well understood. The criteria are extracted from literature or elicited from stakeholders or experts. Another set of approaches starts from observations of presence and/or absence of success and uses these to investigate the factors influencing the occurrence. The emergence and availability of an increasing number of geo-referenced household surveys providing information on existing or wished adoption offers new opportunities to understand determinants of adoption, as well as the spatial dimensions of adoption. A typical approach is to apply probit or other econometric models to these datasets to examine the statistical significance of household and socio-economic variables on the adoption of new practices (Rosenstock et al., 2015). Several authors have gone a step further and created adoption maps. They based them on methods such as small area estimation (Ghosh and Rao, 1994, Haughton and Haughton, 2011) or spatial allocation models (Tatem et al., 2015).

#### 2.1.4 Step 3: Identification of the recommendation domains and out-scaling potential of the options

Where the previous step includes the identification of the criteria that influence the suitability and adoption of the options, this step aims at mapping the places where these characteristics apply, i.e. where the options are likely to be suitable and taken up. We call these recommendation domains. Matching the conditions favouring the successful implementation of options with a spatially referenced (GIS) database, yields the geographical delineation of the recommendation domain. This process implies transforming the previously identified characteristics for a technology into variables for which spatial data exist. The growing popularity of GIS and spatial analysis over the last two to three decades has resulted in a variety of global and regional datasets that are easily and cheaply available from a variety of sources. This is especially true for bio-physical data, with slower progress made in the availability of socio-economic data. The emerging wealth of geo-referenced farm or household level information in combination with standardized representative data, such as census data, allows for the out-scaling of socio-economic variables. Such data is, however, scarce, with USAID's Demographic and Health Surveys (DHS - http://dhsprogram.com/What-We-Do/GIS.cfm) as a welcome but -until now- under-exploited exception. Also local relevance and spatial resolution, remain important challenges. But, with increasing information technology and worldwide data networking, the opportunities to use relevant and good quality data sets are likely to increase further.

Two fundamentally different approaches exist for combining the geographical layers into recommendation domains: approaches based on similarity analysis and approaches based on threshold definition. Similarity analysis approaches make use of geo-referenced observed successes on the ground, link those with the available geographical layers, and identify locations where similar combinations of these geographical variables can be found. Several methods exist, from very simple concepts such as Euclidian or Mahalonobis distances to more complex approaches such as Bayesian network models (Kemp-Benedict, 2008, Kemp-Benedict et al., 2013), multivariate environmental similarity surface (Pfeifer and Omolo, 2014, Zurell et al., 2012) or maximum entropy (Phillips et al., 2006). Threshold-based approaches define thresholds for the different geographic layers based on any of the methods described in Step 2. This approach has been widely used for the delineation of development domains (Chamberlin et al., 2006, Notenbaert et al., 2012) and for spatial targeting (MacAlister et al., 2012, Notenbaert, 2009). The first approach is quite data intensive and requiring evidence of success on the ground. The use and interpretation of the resulting maps is not straightforward. They show a continuous gradient from low to high similarity, but it is often unclear in which direction and to which extent the different factors contribute to this similarity. Also the link between the level of similarity and actual suitability or adoption potential is not always clearly defined. The second approach also works in data poor environments or in locations where evidence from the ground might not reflect success or suitability due to policy distortion. As it is based on known thresholds, the use of the resulting maps is intuitive and they are easy to interpret.

The mapping of recommendation domains can be done in relation to current conditions. When planning with longer timeframes in mind, future projections of the suitability criteria allow the mapping of recommendation domains under scenarios of future change. Finally, this information needs to be complemented with household-level information to match interventions to specific types of producers, as significant heterogeneity exists in farming styles and objectives, resource endowments and farm types within a certain region (Solano et al., 2000, Giller et al., 2011).

#### 2.1.5 Step 4: Ex-ante impact assessment of the alternative options

Information about the nature of the potential impacts, in terms of both the types of impact and their size, is crucial for comparing and prioritising the alternative options. The power of the ex-ante impact assessment results is to be able to explore ‘what ifs’ and to enable actors to engage in a deeper discussion around the potential impacts and trade-offs (Kristjanson et al., 2009). It is thereby important to consider both the temporal and spatial scales of the impacts. Certain interventions may increase productivity in the short term, but in the long run reduce it as an effect of the alteration of key supporting/regulating ecosystem services. An example of this is the introduction of Napier grass in small-holder dairy systems. Planting and harvesting this high-yielding and nutritious grass will in the short run improve diets for the dairy cows and thus increase their productivity. If the grass is being harvested year after year without sufficient inputs of nutrients and organic matter this can lead to localised but extensive soil degradation. In the long term, this loss of soil fertility could overturn the initial success. Similarly, negative impacts can be generated in one place as results of interventions that took place and generated benefits in another place. The upstream/downstream competition for water is a typical example of this. Another element that has to be considered in relation to the impacts of adopted interventions are the trade-offs between objectives. An increase in dairy cow productivity, for example, is leading to higher milk production but at the same time to higher amounts of methane produced by these cows. In addition, the improved productivity and associated income generating opportunity might motivate farmers to keep a larger dairy herd. Although it is expected that the GHG emissions per litre of milk will reduce, this larger number of animals is likely to lead to higher overall GHG emissions (Herrero et al., 2013). The overall assessment thus requires scoring options along the three CSA objectives, productivity/food security, adaptation and mitigation. In addition, supporting CSA decisions requires not only that potential impacts related to these objectives are assessed but also that trade-offs among conflicting objectives are identified and well-communicated. Several methods have been developed in response to this challenge. One notable example is the TOA model as described by Stoorvogel et al. (2004a). TOA constructs trade-off curves that describe the system's inherent relationships between pairs of sustainability indicators. The impact of technological interventions can be assessed by changing appropriate model parameters and then repeat the series of simulations to construct a new set of trade-off curves. The impact of that technology can then be visualized as a change in the trade-off curves. Other methods involve assigning weights to objectives for an additive multiple-objective preference model or multi-objective optimisation.

The exact objectives and goals of the CSA interventions will depend on the context in which they are carried out. These objectives are defined by a set of metrics that we use to measure and evaluate CSA. Several groups, such as the CGIAR Research Program on Climate Change, Agriculture and Food Security (CCAFS) and the World Bank, have undertaken a lot of work to define and elaborate appropriate metrics of CSA. A first sub-step in the ex-ante assessment step thus involves selecting relevant metrics for the envisioned interventions and selected investment region. The diversity of indicators and the varying opinions about their usefulness and importance reinforce the need to select these indicators with end users in a participatory process so that they reflect their goals (Staiger-Rivas et al., 2013). It is important to differentiate the assessment of these metrics by the different groups of people who are likely to be affected by the output of the intervention.

Such an assessment is a very challenging task, and modeling has proven to be a useful tool enabling ex-ante assessment of all possible changes. Connor et al. (2015), for example, argue that it is one of the best means to deal with system complexity and its riskiness and a good way of exploring trade-offs. They find that, in combination with participatory approaches, it effects a positive involvement and interaction of stakeholders and that it adds significant strength and efficiency to designing interventions in agricultural systems. All models are, however, selective and represent a specific simplification of the –often poorly understood- complexity of the real world. The choice of models, as well as their further development and refinement, is thus of utmost importance. By taking a more bottom-up approach to model development that involves stakeholders from the outset, it may be possible to identify and prioritise the problems that need to be solved first, and use this to determine the scope and choice of models to apply (Prell et al., 2007). In order to produce a model that accurately represents the context-specific context and answers, it is necessary to carry out an iterative process of gradual improvement of the model. Connor et al. (2015) further reason that it's important to link model development and validation to data collected from experiments so that data quality can be ensured. It is also very useful to document lessons learned and in doing so inform future decision-making. A notable effort in this realm is the “CSA compendium” initiative that aims to compile a comprehensive database on the impact of field-level agricultural practices on food production, adaptive capacity, and climate change mitigation (Rosenstock et al., 2016).

Also more qualitative methods can be applied. Shikuku et al. (2015), for example, use a simple scoring of indicators and stakeholders' perceptions of the impacts of adaptation options for prioritising adaptation action in Northern Uganda.

### 2.2 Two case studies about livestock development in sub-Saharan Africa

We apply the framework to two example cases and illustrate how targeting in combination with mapping out-scaling potential can be linked to impact assessment and as such contribute to priority setting. The case studies were specifically selected to show the applicability of the framework to very different situations. They cover different scales (local, sub-national vs. regional) and apply a wide range of data collection and analysis methods.

Both cases pertain livestock system development in sub-Saharan Africa. Livestock and livestock production serve multiple functions in millions of peoples' livelihoods. Worldwide, the livestock sector supports about 1.3 billion producers and retailers and contributes 40–50% of agricultural GDP (Herrero et al., 2016). Livestock provide food for at least 830 million food insecure people (Gerber et al., 2010). Animal source foods (ASF) are moreover a critical component of a balanced diet, and contribute to nutrition security in particular of children (Randolph et al., 2007). About 60% of the global cropping area receives manure application (Herrero et al., 2010). The demand for meat and milk is rising quickly, particularly in rapidly expanding urban areas (Alexandratos and Bruinsma, 2012). Globally, the demand for ASF is expected to double by 2050. Considering this rapidly increasing demand for animal source foods, the continued importance of livestock production systems becomes evident; not only for food and nutrition security also for providing livelihood opportunities. The livestock production systems in SSA are characterized by low productivity. In addition, it is predicted that climate change will have significant negative impacts on these livestock production systems (Thornton et al., 2009). At the same time they are the source of considerable greenhouse gas (GHG) emissions (Gerber et al., 2013).

The first example focuses on smallholder mixed crop-livestock systems in the eastern African region. Livestock productivity in these systems is very low. Against the need to increase the productivity of livestock productions systems, are the often negative impacts of climate change on productivity (Thornton et al., 2009). In many places, long-term changes in average temperatures, precipitation, and climate variability threaten livestock production, and thus the food security and the livelihoods of the poor. The livestock production systems in the eastern African region are demonstrating among the lowest GHG efficiencies in the world (Herrero et al., 2013). Tackling climate change has thus become tremendously urgent both from an adaptation and mitigation perspective. In this example we are therefore specifically looking for appropriate “win-win strategies”, contributing to both mitigation and food security objectives.

In the second example, we zoom into the district of Lushoto, a mountainous area in Northern Tanzania. The gap between milk demand and local supply in Lushoto is very large and is projected to continue to widen in the near to medium future. This unmet demand presents an important opportunity for improving the welfare of producers and their market agents, through income and employment generated in dairy production, processing and marketing (Katjiuongua and Nelgen, 2014). In this context the CGIAR Research Program on Livestock and Fish embarked on an effort to transform the Tanzanian dairy value chain (VC) to produce more milk by and for the poor. But efforts to maximize milk yields, production and profitability need to be balanced with long-term sustainability and environmental stewardship. In this case study, we assess the climate-smartness of four best-bet intervention scenarios for adaptation to climate change and increased milk production: (i) introduction of improved breeds, (ii) reduced seasonality of feed availability (iii) improved animal health, (iv) a package of these three technology interventions combined. Through providing rapid results and flagging productivity, adaptation and mitigation issues and challenges simultaneously, this case study aims to support evidence-based discussions of climate-smart development pathways in the Lushoto dairy value chain.

## 3 Results

### 3.1 Overview

Table 1 summarizes the key differences in scales, objectives and approaches used in the case studies. Apart from cutting across scales, the case studies represent applications of the generic framework in different systems and providing information for different end-users, envisioning their own specific and quite varied objectives. These differences result in the choice of a variety of methods and approaches for the analysis stages.

### 3.2 Livestock production in eastern Africa

#### 3.2.1 Diagnosis and identification of potential options

In the eastern African case study, we opted for a data-driven quantitative approach for the first step. The selection of priority areas for action was based on a combination of factors: (i) the relative contribution of different livestock production systems to overall livestock-related GHG emissions and (ii) the level of exposure to climate change. For the first factor we used the global livestock dataset produced by Herrero et al. (2013). We extracted maps and summary statistics in terms of both GHG emissions and emission intensities. In combination with maps of the Standardized Precipitation-Evapotranspiration Index (ILRI, 2014), they revealed that the mixed dairy system is the highest GHG emission source in the region and that it is at the same time highly exposed to drought and climate variability (ILRI, 2014, Gerber et al., 2013). The mixed dairy system was therefore seen as the priority system. Consecutive expert consultations yielded the following promising win-win options for these systems: (i) improved feed quality (consisting of an improvement in feed digestibility through the process of feed residues and the introduction of maize to the ration); (ii) improved animal husbandry and health; and (iii) improved grassland management, in terms of both changing grazing management and increasing legumes in grasslands.

#### 3.2.2 Characterisation of options

Experts selected the win-win adaptation-mitigation strategies while explicitly having the mixed dairy production systems of East-Africa in mind. They were all assumed to be suitable for these systems across the region and expected to increase livestock productivity and at the same time reduce GHG emission intensity. Their adoption potential, on the other hand, was considered to be associated with different key drivers. Access to markets, or more specifically ‘travel time to cities’, was chosen as proxy for adoption of the option ‘improved feed quality’, assuming that farmers closer to the market are more likely to adopt new technologies (Silvestri et al., 2012, Maddison, 2007, Solano et al., 2000). For the option ‘improved animal husbandry and health’ we used the proxy ‘rural population density’ considering that the health of animals is affected by nutrition, and by external factors such as stress reduction and preventative health measures like vaccinations, and assuming that the proximity to more densely populated area would ensure the access to for example veterinary services. Pulse yield was used as a proxy for ‘improved grassland management’. Indeed, one of the most common grassland management options is oversowing (Lucas et al., 1980, Macfarlane and Bonish, 1986), i.e. legumes into existing pasture. We therefore assumed that stakeholders are more likely to adopt this option in areas suitable for the growth of pulses.

#### 3.2.3 Identification of the recommendation domains and out-scaling potential of the options

The geographical boundaries of relevant livestock production systems were extracted from the maps produced by Robinson et al. (2011). The recommendation domains for all three options were considered to be inside the mixed crop-livestock systems. The grassland management option was further narrowed down and considered to only be applicable in the grazing lands as defined by Ramankutty et al. (2008). Within these areas, the factors hypothesized to influence adoption were mapped out and converted into a normalised index ranging from 0 to 1. The resulting map range represents the fraction of farmers that is likely to adopt the strategy, i.e. one implies that every farmer in that specific location is likely to adopt the strategy, zero that none of the farmers is likely to adopt. Multiplying this fraction with the number of dairy farmers in the region, provides a first quantification of out-scaling potential, i.e. the number of farmers potentially adopting the options within the recommendation domains.

#### 3.2.4 Ex-ante impact assessment of the alternative options

In addition, the alternative options were modelled with the Global Livestock Environment Assessment Model (GLEAM). This tool enables the production of disaggregated estimates of GHG emissions and emission intensities for different farming systems within selected regions (Gerber et al., 2013). Two of the three CSA pillars were modelled with GLEAM:

1) Productivity pillar: the envisioned increase in productivity is believed to support food and nutrition security. The selected indicator is:
  a. Milk supply (kg Fat-Protein Corrected Milk)
2) Mitigation pillar: the selected indicators include total emissions and emission intensities:
  a. GHG emissions (kg CO_2_-equivalent)
  b. GHG emission intensity (kg CO_2_-equivalent/ha, kg CO_2_-equivalent/kg FPCM)

#### 3.2.5 Implications for research and development

With feasible improvements in feed quality, animal health and husbandry, and grassland management, a reduction of 5% to 24% of baseline emissions can be achieved in mixed diary systems in East Africa. All the explored mitigation options can concomitantly lead to a reduction of emission intensity and an increase in production. Diet improvements through improved digestibility have the highest mitigation potential. The mitigation potential when holding milk production constant largely results from a reduction in animal numbers, since yield gains allow to achieve the same milk production with fewer animals. An emission reduction of 10% to 24% is expected for mixed diary systems in East Africa, when holding output constant. When holding the number of adult female animals constant, and thus increasing the output, the absolute mitigation potential is lower than when output is held constant. Nonetheless, the options result in a decrease in overall emissions of 5% to 10% in combination with an increase in milk production ranging from 6% to 18% (Mottet et al., 2016).

Improving efficiency and reducing the gap in emission intensity could be thus be achieved by improving existing practices.

This assessment does not include considerations about possible barriers to adoption. Interventions should be carefully designed and targeted, and this requires a better understanding on how to out-scale the results obtained from individual trials. Institutional, economics and behavioural barriers are all likely to play a major role in the uptake of these practices (García de Jalón et al., 2014). Furthermore, other information would need to be integrated in the planning of wide scale application of the mitigation options, such as more broad environmental impact assessments and animal welfare considerations.

### 3.3 Dairy VC development in Lushoto, Tanzania

#### 3.3.1 Diagnosis and identification of potential options

The diagnosis and identification of alternative intervention scenarios for the dairy value chain in Lushoto was purely based on participatory consultations with a variety of VC actors.

During a multi-stakeholder workshop in Lushoto it was agreed that livestock keeping falls in three broad categories: (i) semi-intensive mixed crop-livestock systems, (ii) extensive agro-pastoral, and (iii) ranching. The ranching system is very rare, with only two known ranches in the region, and typically geared towards beef production. It was thus excluded from further analysis. In addition, sixteen village-level innovation platforms (IPs) were established. These IPs bring together individuals with different backgrounds and interests, such as farmers, traders, food processors, researchers, government officials, etc. and provide a useful space for local stakeholders to jointly identify constraints and devise and implement solutions. Each of the IPs came up with a site-specific plan that includes recommendations for VC transformation. These recommendations didn't take the potential environmental impacts into account, but were selected on the basis of stakeholders' best estimates about their potential economic and livelihood impacts. We examined the sixteen village plans and found that all proposed interventions fell in four general intervention scenarios: (i) introduction of improved breeds, (ii) reduced seasonality of feed availability, (iii) improved animal health, (iv) a package of these three technology interventions combined.

#### 3.3.2 Characterisation of options

All dairy VC intervention scenarios were expected to be applicable in both the semi-intensive mixed crop-livestock systems and the extensive agro-pastoral systems. They were first characterized through focus group discussions. This characterisation was validated and refined through expert opinion interviews and literature review. Each of the intervention scenarios was described in terms of expected changes in milk yield, feed baskets, livestock body weight, and herd size and composition. These changes were expected to be different for the different production systems. The genetic scenario was, for example, assumed to play out in the mixed crop-livestock systems through increased live weight of cattle, but restricted milk yield increase due to the limiting effects of production diseases such mastitis and infections. Within the extensive system an improvement of genetics providing increases in the milk yield per animal was assumed to go hand-in-hand with a reduction of herd size to compensate for increased nutritional requirements and decreasing resilience due to factors such as temperature affecting disease pressure and reproductive functions of the animals.

#### 3.3.3 Identification of the recommendation domains and out-scaling potential of the options

We performed a participatory mapping exercise, resulting in the geographical delineation of the different livestock production systems (Morris et al., 2014). About thirty one thousand livestock enterprises of the semi-intensive mixed crop-livestock type were estimated to jointly produce about 30% of the milk in the region. The nine thousand or so extensive agro-pastoral livestock enterprises on the other hand were approximated to account for about 70% of the milk production in the region. We assumed a single adoption rate of 20% for all options, a percentage that is within the range of observed adoption of technologies in the East-African Dairy Development program (Kiptot et al., 2015). We thus assumed that the total number of enterprises wouldn't be changing but that 20% of them would adopt the respective intervention scenario, while the other 80% would not make any changes. The potentially increased milk supply was assumed to be fully absorbed by the market.

#### 3.3.4 Ex-ante impact assessment of the alternative options

In this case-study we covered the three CSA pillars as follows:

3) Productivity pillar: the envisioned productivity increases are meant to support food and nutrition security. Selected indicators under this pillar are:
  a. Milk supply (kg Fat-Protein Corrected Milk)
  b. Productivity (kg FPCM/ha)
4) Adaptation pillar: indicators selected for reflecting resource-use efficiency, resilience and adaptive capacity are:
  a. Soil loss (kg, kg/ha, kg/kg FPCM)
  b. Nitrogen balance (kg, kg/ha, kg/kg FPCM)
5) Mitigation pillar: the selected indicators include not only total emissions but also emission intensities:
  a. GHG emissions (kg CO_2_-equivalent)
  b. GHG emission intensity (kg CO_2_-equivalent/ha, kg CO_2_-equivalent/kg FPCM)

**Fig. 2:**
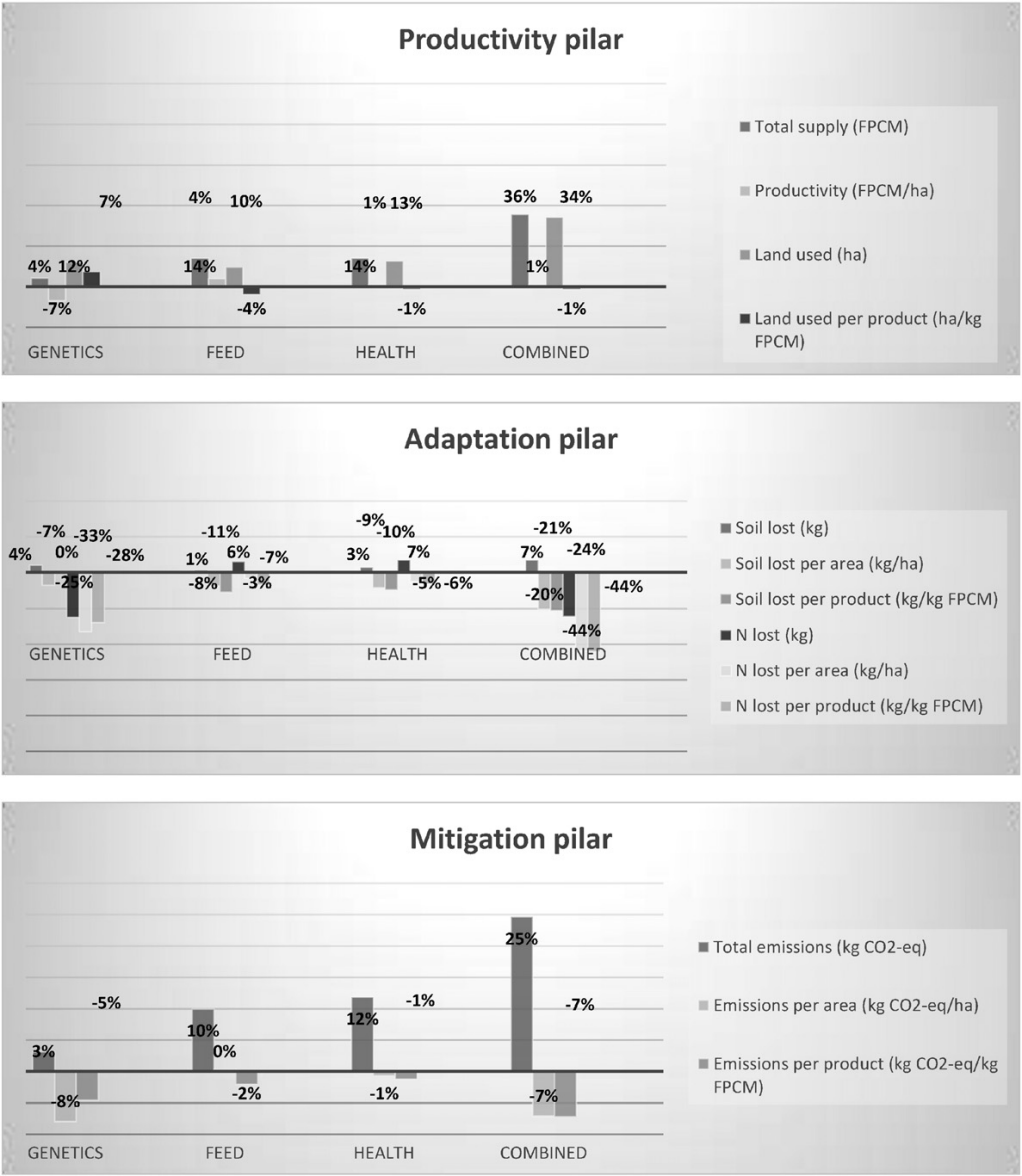
Projected changes in productivity, adaptation and mitigation indicators in the dairy VC in Lushoto under the different scenarios.

Fig. 2 shows the projected changes in each of these indicators under the different scenarios.

In a first step, local farm-level impacts were estimated. It was found that total milk supply would go up under all scenarios, but mostly so if the genetic, feed and animal health interventions were packaged together. The biggest supply gains would be made in the mixed crop-livestock farms. These productivity increases are expected to go hand-in-hand with increased natural resource use but improved resource-use efficiency. Also an overall rise in GHG emissions is expected. In contrast to the expectation of higher total GHG emissions, the models mostly project lower emission intensity, especially when expressed per product. Due to the current low productivity of the agro-pastoral dairy herds, highest gains in efficiency in combination with relatively low increases in total GHG emissions are expected in these types of enterprises.

The assumption made for aggregating the local impacts into regional impacts is that each of the scenarios are likely to have the same relevance for areas falling within the same livestock production system and that the projected impacts can be widely applied across the landscape, region or country. Regional impacts were thus calculated based on the estimated level of adoption and the importance of each of the systems.

#### 3.3.5 Implications for research and development

The investigated productivity-enhancing interventions would mostly result in mitigation co-benefits across the different systems. The only exception is the improved genetics scenario in the mixed farms. Also in relation to the adaptation and food security indicators, the genetics scenario might lead to negative effects due to the likelihood that herd sizes would decline, reducing productivity in both production systems. Moreover, soil and N losses are projected to increase significantly in the agro-pastoral systems. The feed and animal health intervention scenarios are also expected to increase N losses in the mixed systems. In these systems it is thus important to re-direct some of the manure to the feed crops for increased long-term sustainability and resilience. Increased nutrient provision while modelled to deliver benefits in the energy restrictive dry season, is self-limited in benefits to milk production and weight gain by breed characteristics and impacts of poor health status. In general, the packaged intervention is estimated to be more climate smart than an isolated one-technology focused approach.

In this case study, the aim was to support evidence-based decision making on development pathways in the Tanzanian dairy value chain. The ex-ante assessment was therefore designed to provide rapid results, so that evidence could be provided in a timely manner. To ensure that the information is relevant and building on stakeholders' understanding of development opportunities, the assessments were applied to intervention scenarios coming out of earlier stakeholder discussions. In addition, they were targeted to production systems equally identified in a participatory manner. The results were shared with stakeholders at the Tanga Dairy platform and will help them to tweak technology-based interventions.

Despite the undoubted benefits of the more sophisticated technology scenarios brought forward by the stakeholders and which the models can compute, rudimentary interventions such as implementing realistic health and husbandry interventions (such as provision of water ad infinitum, better designed housing and udder hygiene) will most significantly improve the health status and thus milk production. A significant reduction in GHG emissions, soil and N loss could be achieved by education in manure collection, storage and subsequent application to cultivated crops. Potentially, the greatest benefit could be provided by implementing these basic interventions at a very low cost.

## 4 Discussion

### 4.1 Out-scaling potential and the importance of understanding adoption

The identification of recommendation domains and establishment of out-scaling potential serves multiple objectives. On one hand, it supports the geographical targeting of options. Maps with option-specific recommendation domains can help development actors to decide which options to promote where. Related assessments and/or maps of out-scaling potential can give an indication if it is worth investing in a certain option at all. In addition, the understanding of determinants and barriers to adoption can point to a new (set of) interventions, addressing the adoption incentives and barriers.

On the other hand, the recommendation domains and out-scaling potential are also important factors feeding into the ex-ante impact assessments. More specifically, if potential impacts of the wide-scale promotion of local actions are to be assessed, the reach or likelihood of adoption of the options has a huge influence on overall impact. It is important to take into account that only a fraction of the population is likely to implement the CSA interventions and to reduce the sizes of the potential target groups accordingly when estimating aggregated impacts. If local models are being linked to global and regional change models, the scenarios run by these larger-scale models equally need to be informed by the assessments of out-scaling potential.

### 4.2 The choice of approach and methods

As can be seen from the examples, a variety of methods can be used when applying the generic framework. Depending on the target users and their objectives, different indicators, methods and scales at which to apply these methods are appropriate.

When informing farmers' decision making processes about their choice between different farming practices, it can suffice to provide comparisons of the options in terms of -for example- expected yield increases and suitability to future climate conditions. To farmers it's often only the impacts at the local level which are of interest. In addition, they might be satisfied with qualitative comparisons only and a simple ranking of options could thus be adequate. Farmers are generally less interested in the out-scaling potential and impacts at the larger scale. As soon as stakeholders from different locations, such as both up- and downstream farmers, are involved, it is however important to provide information beyond the local level. In this case, the interaction between mapping recommendation domains, assessing out-scaling potential and ex-ante assessments at different scales becomes of higher importance. Often a combination of different methods, linked in a more or less systematic way, is needed. It is thereby important to note that, depending on the spatial scale, more or less details and complexities will need to be represented by the models. Development actors, such as governmental and non-governmental organizations, would equally be interested in potential impacts at both local and broader scales. Of specific interest to them will be the assessments of recommendation domains and out-scaling potential. Policy makers and policy analysts in charge of drafting new regional or national policies, on the other hand, are often looking for aggregate numbers. In that case, quantitative methods will have to be applied. In terms of scale, however, they are often interested in the larger scale only, with much less interest in the geographical heterogeneity underlying the aggregate numbers.

Also the expected time-frame within which decisions can be influenced is of paramount importance when making methodological choices. In the case of the dairy VC transformation in Lushoto, for example, development actors are at the verge of making important investment decisions. Through providing rapid results and flagging the main issues, we aim to support evidence-based discussions of alternative CSA- ready development pathways in the Tanzanian dairy value chain, a concept that would otherwise have been completely overlooked.

Another time-related aspect to take into account is the temporal scale of the assessments. It is clearly important to incorporate assessments of potential impacts in the future, and as such provide evidence about the alternative options' longer-term sustainability. Our rapidly changing world also necessitates the testing of the options' suitability, adoptability and impacts under different scenarios of future change and variability. This provides some evidence about the options' robustness, which could be an important consideration to be taken into account by the decision makers.

### 4.3 Stakeholder involvement

The aim of the evidence produced in both examples is to influence decision-making and eventually change agricultural practices on-the-ground. There are many stakeholders as well as policy levels that are involved in agricultural systems and the interests of all these groups can be quite diverse. Participatory approaches are powerful ways to identify actor-relevant objectives and indicators and come up with intervention scenarios (Klapwijk et al., 2014). Other authors add that participatory approaches allow to tap into local knowledge and to validate the feasibility of potential options (Stoorvogel et al., 2004b, Giller et al., 2011, Vayssieres et al., 2007). The early involvement of stakeholders raises awareness and creates support for the issue and its solutions. As such, it reduces the risk of the recommendations not being carried out. The process for producing the evidence is thus often at least as important as the actual information produced. Going through the multi-stage proces with stakeholders in an iterative way encourages learning and consequential refining of analysis and results. This process allows for an increasingly deeper understanding and increasing levels of trust and buy-in from stakeholders.

The role the stakeholders play through the participatory involvement does change with scale. In the dairy example in Lushoto, the involvement of livestock keepers and other actors along the dairy value chain is meant to ensure the inclusion of views of stakeholders with real decision power in the dairy VC. This will, hopefully, lead to the choice of sustainable practices on-farm or further along the VC. The example at regional scale, on the other hand, aims at influencing higher level policies and engagement with farmers was limited.

As agricultural systems and their links with markets and interactions with the wider context are very complex, cross-disciplinary analysis is often required. In such cases, an added advantage of involving a wide variety of stakeholders is that access to relevant knowledge, which is typically distributed among several stakeholders, can be assured. The accuracy of data provided by local stakeholders might be of limited geographical scope. At larger scales, the role of experts for information provision therefore increases.

## 5 Conclusion

Ideally, decision-making processes are evidence-based and informed by objective information. The provided evidence should assist in narrowing down long lists of potential solutions into real portfolios of context-specific action. The framework presented in this paper provides a generic, step-by-step guide for supporting this process. It has been illustrated with examples across a variety of livestock production systems and scales that the generic framework is indeed applicable in a different forms and settings. The process of generating the data in a more or less participatory manner, has been shown to influence the buy-in of the decision-makers and thus the eventual impact.

It is, however, important to note that the data, knowledge and information that go into the process ultimately determine the quality of the resulting evidence and thus the eventual decisions made. Firstly, further research leading to a greater understanding of barriers and incentives to technology uptake and adoption is thus urgently needed. Secondly, further investment and effort should be directed to the creation of additional and more accurate spatially disaggregated data about variables that influence suitability and impacts of interventions in agricultural systems. Lastly, a greater understanding of all processes and interactions at different scales will increase the quality of models and robustness of ex-ante impact assessments.

## Figures and Tables

**Table 1:** Summary of scale, objectives and approaches used in the case studies.

	Climate-smart livestock production in eastern Africa	Transformation of the dairy VC in Lushoto district
Scale	Regional	Local
System under study	A variety of livestock production systems	Dairy value chain (VC)
Objectives	Climate change mitigation and food security	Climate-smart dairy VC development
Next users	Policy makers at global and regional level	Village and district-level dairy innovation platforms (IP)
Diagnosis	Mapping of regional livestock production systems, GHG emissions and SPEI index	Discussion at village and district level IPs
Identification of alternative options	Expert opinion	Discussion at village and district level IPs
Characterisation of options	Expert opinion	Focus group discussions, expert opinion and literature review
Map recommendation domains and out-scaling potential	GIS	Participatory GIS
Ex-ante impact assessment	Global modeling (GLEAM)	Farm-scale modeling (nutrient balances and GHG emissions)
